# A Soluble Epoxide Hydrolase Inhibitor Improves Cerebrovascular Dysfunction, Neuroinflammation, Amyloid Burden, and Cognitive Impairments in the hAPP/PS1 TgF344-AD Rat Model of Alzheimer’s Disease

**DOI:** 10.3390/ijms26062433

**Published:** 2025-03-08

**Authors:** Xing Fang, Jane J. Border, Huawei Zhang, Lavanya Challagundla, Jasleen Kaur, Sung Hee Hwang, Bruce D. Hammock, Fan Fan, Richard J. Roman

**Affiliations:** 1 Department of Pharmacology and Toxicology, University of Mississippi Medical Center, 2500 North State Street, Jackson, MS 39216, USA; xfang@umc.edu (X.F.); jjryu@umc.edu (J.J.B.); hzhang2308@gmail.com (H.Z.); 2 Molecular and Genomics Facility, University of Mississippi Medical Center, Jackson, MS 39216, USA; lchallagundla@umc.edu (L.C.); jkaur4@umc.edu (J.K.); 3 Entomology and Nematology and UC Davis Comprehensive Cancer Center, University of California Davis, Davis, CA 95616, USA; shhwang@ucdavis.edu (S.H.H.); bdhammock@ucdavis.edu (B.D.H.); 4 Physiology, Medical College of Georgia, Augusta University, Augusta, GA 30912, USA; ffan@augusta.edu

**Keywords:** dementia, Alzheimer’s disease, soluble epoxide hydrolase, neurovascular coupling, capillary rarefaction, blood–brain barrier, neuroinflammation

## Abstract

Alzheimer’s disease (AD) is an increasing global healthcare crisis with few effective treatments. The accumulation of amyloid plaques and hyper-phosphorylated tau are thought to underlie the pathogenesis of AD. However, current studies have recognized a prominent role of cerebrovascular dysfunction in AD. We recently reported that SNPs in soluble epoxide hydrolase (sEH) are linked to AD in human genetic studies and that long-term administration of an sEH inhibitor attenuated cerebral vascular and cognitive dysfunction in a rat model of AD. However, the mechanisms linking changes in cerebral vascular function and neuroprotective actions of sEH inhibitors in AD remain to be determined. This study investigated the effects of administration of an sEH inhibitor, 1-(1-Propanoylpiperidin-4-yl)-3-[4-(trifluoromethoxy)phenyl]urea (TPPU), on neurovascular coupling, blood–brain barrier (BBB) function, neuroinflammation, and cognitive dysfunction in an hAPP/PS1 TgF344-AD rat model of AD. We observed predominant β-amyloid accumulation in the brains of 9–10-month-old AD rats and that TPPU treatment for three months reduced amyloid burden. The functional hyperemic response to whisker stimulation was attenuated in AD rats, and TPPU normalized the response. The sEH inhibitor, TPPU, mitigated capillary rarefaction, BBB leakage, and activation of astrocytes and microglia in AD rats. TPPU increased the expression of pre- and post-synaptic proteins and reduced loss of hippocampal neurons and cognitive impairments in the AD rats, which was confirmed in a transcriptome and GO analysis. These results suggest that sEH inhibitors could be a novel therapeutic strategy for AD.

## 1. Introduction

More than 55 million people worldwide are affected by dementia, with Alzheimer’s disease (AD) accounting for 60–70% of the cases [1]. Seven million people in the United States live with AD [2]. Between 2000 and 2021, while deaths from heart disease decreased by 2.1%, deaths from AD rose by 141% [2]. Despite this staggering statistic, only nine drugs have been approved by the FDA to treat AD. Six of these agents focus on alleviating cognitive and behavioral symptoms without affecting the underlying pathological changes. The other three drugs, aducanumab, lecanemab, and donanemab, are anti-amyloid monoclonal antibodies that effectively reduce the burden of amyloid in the brain and can potentially delay disease progression [2,3]. However, the results of these treatments in reversing cognitive dysfunction have been disappointing in that only lecanemab and donanemab have exhibited a modest benefit in clinical trials [4,5]. Moreover, amyloid monoclonal antibody therapy is costly, and up to one-third of patients still develop amyloid-related imaging abnormalities (ARIAs), which has led to the discontinuation of aducanumab from the market [5,6].

Recent studies have recognized the prominent roles of cerebrovascular dysfunction, neuroinflammation, and hypoperfusion in AD [7,8,9]. Advanced imaging techniques have revealed that AD is associated with reduced resting cerebral blood flow (CBF) [9,10,11,12], impaired functional hyperemia [12,13,14], blood–brain barrier (BBB) dysfunction [15,16,17,18,19], and alterations in microvascular morphology [13]. Subsequent activation of astrocytes and microglia release proinflammatory cytokines (interleukin-1β, tumor necrosis factor-α, interleukin-6) and chemokines that also contribute to a “neuroinflammatory milieu,” leading to synaptic dysfunction, neuronal damage, and further Aβ deposition that accelerates disease progression [20]. However, the mechanisms involved remain poorly understood, and no agents have been developed that specifically address cerebrovascular dysfunction in AD. Moreover, clinical trials of drugs targeting neuroinflammation have failed to exhibit the expected clinical efficacy [21].

Long-chain polyunsaturated fatty acids such as arachidonic acid (AA) are metabolized by cytochrome P450 enzymes to epoxyeicosatrienoic acids (EETs) and other epoxy-fatty acids (EpFAs) [22]. EETs and EpFAs are potent vasodilators and reduce inflammation and oxidative stress [23]. EpFAs are rapidly metabolized by soluble epoxide hydrolase (sEH) to their corresponding diols, thus limiting their beneficial inflammatory-resolving effects [24,25]. sEH levels are elevated in the brains of AD patients and transgenic animal models [26,27,28,29]. In human genetic association studies, SNPs in sEH have been linked to AD [30]. sEH is highly expressed in astrocytes and the vasculature [26,27,31,32]. Inhibitors of sEH have been reported to attenuate neuroinflammation in mouse models of AD [26,27,28,33].

We recently reported that long-term administration of a highly selective inhibitor of sEH, N-[1-(1-oxopropyl)-4-piperidinyl]-N’-[4-(trifluoromethoxy) phenyl)-urea (TPPU) attenuated the loss of cognitive function and impaired myogenic response and autoregulation of CBF in the hAPP/PS1 TgF344-AD rat model of AD [34]. However, the mechanisms linking the improvements in cerebral vascular function and the neuroprotective actions of sEH inhibitors in AD remain to be determined. The present study evaluated the effects of TPPU on amyloid pathology, neurovascular coupling (NVC), BBB integrity, neuroinflammation, loss of neurons, and cognitive function in the hAPP/PS1 TgF344-AD rat model of AD. An RNA sequencing study and Gene Ontology analysis were also performed to explore the potential mechanisms involved.

## 2. Results

### 2.1. The Expression of sEH Is Elevated in AD Brains

**Figure 1:**
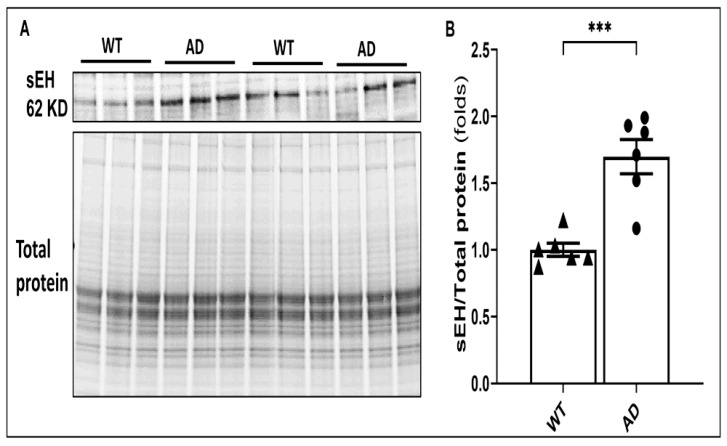
Elevated expression of sEH in AD rats. (**A**) Representative Western blot comparing the expression of 62KD sEH band in whole brain tissue from 9-month-old APP/PS1 TgF344-AD (AD) rats (n = 6) and age-matched Fischer 344 (WT) animals (n = 6). (**B**) Quantification of the expression of the 62 KD sEH from panel A was normalized by the sum of the total protein bands detected per lane on a stain-free gel. Data are presented as means ± SEM. *** *p* < 0.001. It should be noted that a prominent cross-reactive band was detected at 37 KD using the sc-166961 monoclonal anti-sEH (A-5) antibody, which is the most commonly used antibody in the field. It has been employed in 25 published Western blot and immunohistochemistry studies (https://www.scbt.com/p/seh-antibody-a-5 (accessed on 27 June 2024)). In some studies, others have observed a 37 KD band using the A5 and other sEH antibodies. Despite our efforts to optimize the protocol or when using other sEH antibodies, we consistently detect the 37 KD band when assaying whole brain tissue homogenates. There is consensus that the 62 KD band is full-length active sEH. The other band has not been identified. It may be a cleavage product or some unknown cross-reactive protein since its expression does not mimic the 62 KD band. Regardless, the presence of this band does not impact the present results based on the expression of the 62 KD full-length sEH protein.

Previous studies have reported that the expression of sEH was elevated in postmortem brains collected from AD patients and transgenic mouse and rat models of AD [27,28]. In the present study, we confirmed that the expression of sEH was significantly enhanced in the brains of 9-month-old APP/PS1 TgF344-AD rats versus F344 WT rats (Figure 1).

### 2.2. TPPU Attenuates Beta-Amyloid (Aβ) Accumulation in AD Rats

**Figure 2:**
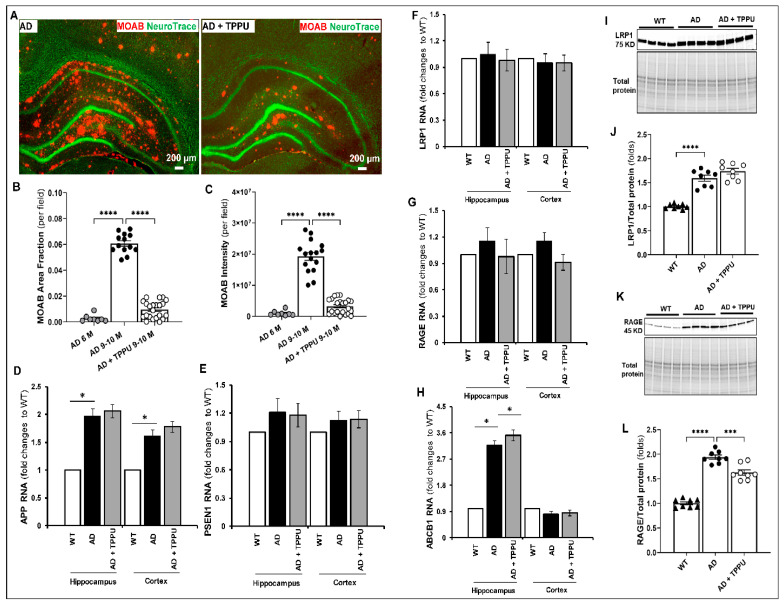
TPPU attenuates amyloid accumulation in AD rats. (**A**) Representative images of co-immunostaining β-amyloid with MOAB antibody and Green Fluorescent NeuroTrace of neurons in the hippocampus and cortex of 9–10-month-old APP/PS1 TgF344-AD (AD, n = 11) and 9–10-month-old AD rats treated with TPPU for 3 months (AD + TPPU) (n = 10). (**B**,**C**) Comparison of the area of amyloid deposition and intensity of staining per field in the hippocampus and cerebral cortex of 6-month-old AD (n = 5), 9–10-month-old AD (n = 10), and 9–10-month-old AD + TPPU (n = 10) rats. (**D**,**E**) Comparison of RNA expression of APP and PS1 (PSEN1) in 9–10-month-old WT, AD, and AD + TPPU rats. (**F**–**H**) Comparison of RNA expression of low-density lipoprotein receptor-related protein 1 (LRP1), receptors for advanced glycation end products (RAGE) and ATP-binding cassette sub-family B member 1 (ABCB1, P-glycoprotein 1) involved in vascular clearance of β-amyloid in the cortex and hippocampus of 9–10-month-old WT, AD, and AD + TPPU rats. The mRNA expression levels obtained from RNA sequence analysis were normalized to mean values determined in the WT group. (**I**,**K**) Representative Western blots of the expression of LRP1 and RAGE proteins in the brain of 9–10-month-old WT (n = 6), AD (n = 6), and AD + TPPU (n = 6) rats. (**J**,**L**) Quantification of the LRP1/total protein and RAGE/total protein expression ratios in the Western blots. The expression of the target proteins was normalized by the sum of the total protein detected per lane on the stain-free gel. The values in individual animals were normalized to the average mean ratio obtained in the WT group. Data are presented as means ± SEM. * *p* < 0.05, *** *p* < 0.001, **** *p* < 0.0001.

The APP/PS1 TgF344-AD rats have been reported to progressively develop Aβ accumulation in the hippocampus and cerebral cortex as they age [35]. The present study found that Aβ plaques first appear at 6 months of age and are markedly elevated in the cortex and hippocampus of 9–10-month-old AD rats (Figure 2A–C). We next examined whether the chronic administration of TPPU attenuated Aβ accumulation in this model. The area and intensity of Aβ plaques were significantly reduced in TPPU-treated AD rats compared to non-treated AD rats (Figure 2A–C). However, the amyloid levels in the brains of 9–10-month-old AD rats treated with TPPU were still higher than those seen in 6-month-old untreated AD rats (Figure 2B,C), suggesting that while TPPU slowed the accumulation of amyloid, it could not reverse the established pathology. We also explored whether the reduced amyloid load was due to synthesis inhibition or enhanced clearance. Our RNA sequencing study found that amyloid precursor protein (*APP*) expression was significantly higher in the cortex and hippocampus in AD animals than in WT rats. TPPU did not alter *APP* or presenillin1 (*PS1*) mRNA expression in the AD rats (Figure 2D,E). We also compared the effects of TPPU on the mRNA and protein expression of the primary receptors and transporters involved in Aβ clearance, such as the low-density lipoprotein receptor-related protein 1 (LRP1), the receptor for advanced glycation end products (RAGE), and P-glycoprotein (also known as ATP-binding cassette sub-family B member 1, (ABCB1) [36]. LRP1, one of the primary receptors responsible for exporting Aβ, was elevated in AD rats at the protein level, and TPPU treatment did not impact the expression (Figure 2F,I,J). The expression of the RAGE that binds Aβ and impedes its clearance was upregulated in AD rats compared to WT rats at the protein but not the RNA level. Additionally, TPPU treatment significantly reduced the expression of RAGE protein in the brains of AD rats (Figure 2G,K,L). The expression of the multidrug resistance protein 1, *ABCB1,* which helps clear Aβ from the brain into the circulation, was 2-fold higher in the hippocampus but not the cortex of AD versus WT rats. TPPU treatment further increased *ABCB1* mRNA levels in the hippocampus of AD rats (Figure 2H). Thus, TPPU attenuated amyloid accumulation in AD rats partly by improving its clearance rather than reducing production.

### 2.3. TPPU Normalizes Functional Hyperemic Responses in AD Rats

**Figure 3:**
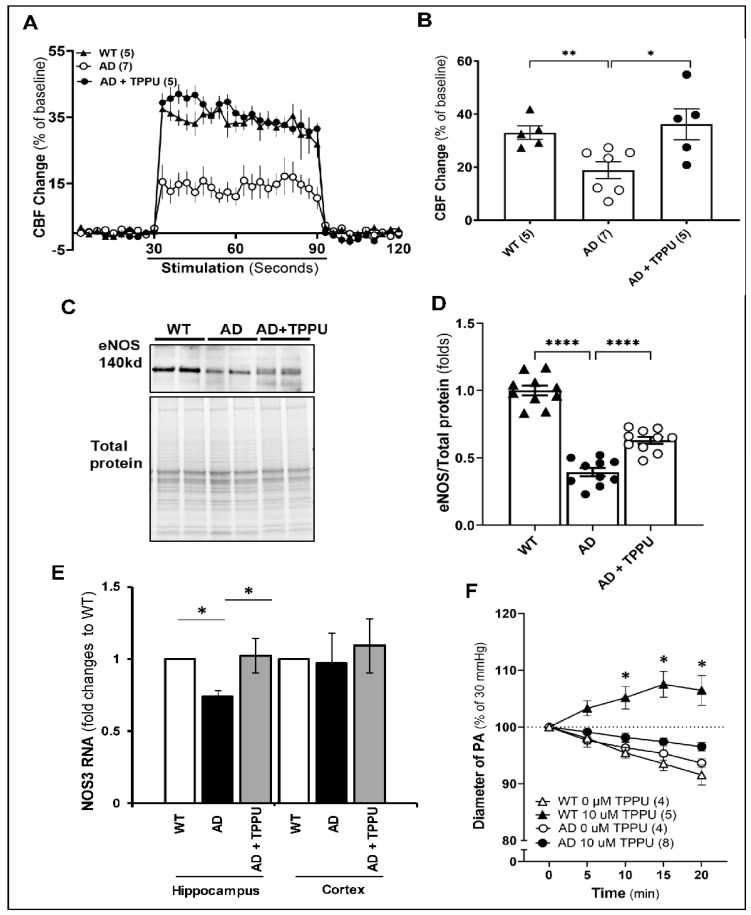
TPPU normalizes functional hyperemic responses in AD rats. (**A**) Comparison of changes in cerebral blood flow (CBF) response to whisker stimulation in 9–10-month-old Fischer 344 (WT) (n = 5), APP/PS1 TgF344-AD (AD) (n = 7), and AD rats treated with TPPU for 3 months (AD + TPPU) (n = 5) rats. (**B**) Comparison of the changes in CBF relative to baseline in the animals from panel (**A**). (**C**) Representative Western blot showing the expression of eNOS in whole brain tissue from 9–10-month-old WT (n = 6), AD (n = 6), and AD + TPPU (n = 6) rats. (**D**) Quantification of eNOS/total protein ratio in the Western blots. The expression of the target proteins was normalized by the sum of the total protein detected per lane on the stain-free gel. The eNOS/total protein ratios in AD and AD + TPPU are normalized to the average values in the WT group. (**E**) Comparison of *Nos3* RNA expression in the cortex and hippocampus of WT, AD and AD rats treated with TPPU. (**F**) Comparison of the response of perfused parenchymal arteriole (PA) isolated from WT and AD rats to short-term administration of vehicle or TPPU (10 µM). Numbers in parentheses represent the number of animals studied. Data are presented as means ± SEM. * *p* < 0.05, ** *p* < 0.01, **** *p* < 0.0001.

We previously reported that the expression of the inward-rectifier potassium (Kir2.1) channel in the cerebral microcirculation, the vasodilator response of the PA to potassium, and the functional hyperemic response to whisker stimulation were reduced in APP/PS1 TgF344-AD rats at 6 months of age [37]. Therefore, as expected, the 9–10-month-old AD rats in the present study failed to redistribute CBF to activated neuronal areas as well as the control rats, and the increase in CBF was only 14% versus 37% in the WT rats. Long-term TPPU treatment normalized the functional hyperemic response to whisker stimulation in the AD rats to the same level seen in the WT rats (Figure 3A,B). In response to elevated neuronal activity, neurons and astrocytes release vasoactive agents that target contractile smooth muscle cells (SMCs) and pericytes to increase local CBF, a process known as neurovascular coupling (NVC) [14]. Nitric oxide (NO) is also a mediator of NVC, and blockade of endothelial nitric oxide synthase (eNOS) typically reduces functional hyperemic responses to whisker barrel stimuli by 50% [38,39]. Therefore, we examined whether the expression of eNOS is diminished in our AD rats and whether TPPU altered eNOS expression. The results indicated that eNOS protein expression was reduced in AD rats’ brains, which was improved by treating these rats with TPPU (Figure 3C,D). The expression of *Nos3* mRNA, which encodes for eNOS, was markedly reduced in the hippocampus of AD compared to WT rats. The levels were restored in AD rats treated with TPPU (Figure 3E). We also tested the vascular reactivity of freshly isolated parenchymal arterioles (PAs) in response to short-term blockade of sEH with TPPU (10 µM) ex vivo. As presented in Figure 3F, TPPU dilated PAs isolated from WT rats but not from AD rats.

### 2.4. TPPU Attenuates Capillary Rarefaction in AD Rats

**Figure 4:**
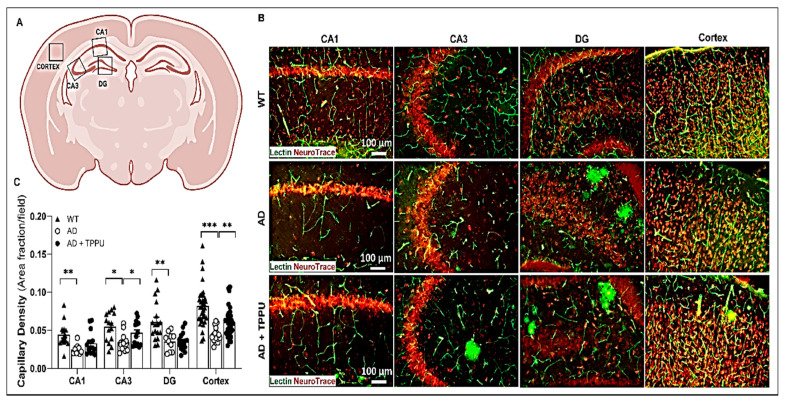

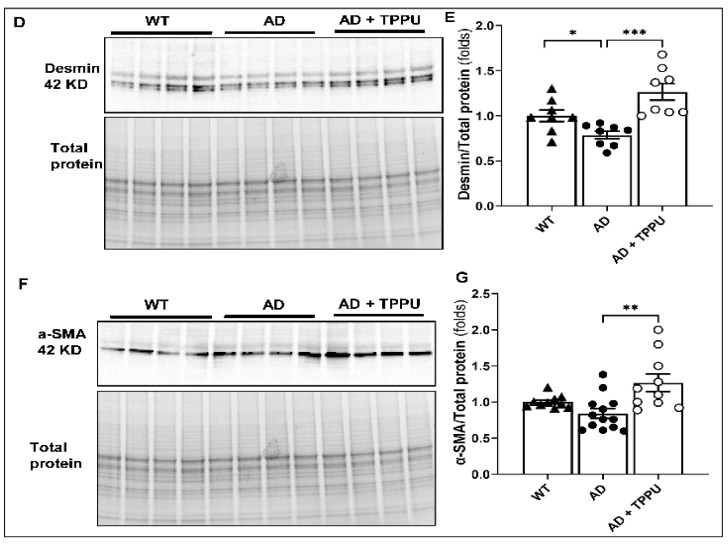
TPPU attenuates capillary rarefaction in AD rats. (**A**) Schematic representation of the regions studied in the cerebral cortex and CA1, CA3, and dentate gyrus (DG) regions of the hippocampus. (**B**) Representative images of co-immunostaining of penetrating arterioles and capillaries with green tomato lectin and neurons with red fluorescent NeuroTrace in hippocampus and cortex of 9–10-month-old Fischer 344 (WT) (n = 11), APP/PS1 TgF344-AD (AD) (n = 10), and AD treated with TPPU for 3 months (AD + TPPU) (n = 11) rats. (**C**) Quantification of the lectin (+) area fraction per field in the CA1, CA3, and DG of the hippocampus and cerebral cortex. (**D**,**F**) Representative Western blots showing the expression of desmin and α-smooth muscle actin (α-SMA) in the brains of 9-10-month-old WT (n = 6), AD (n = 6), and AD + TPPU (n = 6) rats. (**E**,**G**) Quantification of the 52 KD Desmin band/total protein expression and the 42 KD α-SMA/total protein ratios in the Western blots. The expression of the target proteins was normalized by the sum of the total protein detected per lane on the stain-free gel. The values in individual animals were normalized to the average expression level in the WT group. Data are presented as means ± SEM. * *p* < 0.05, ** *p* < 0.01, *** *p* < 0.001. The detection of the lower bands in the desmin Western blot has been a common finding using many antibodies, and has been attributed to secondary structure and folding, which enhances migration of desmin in the gel. When these bands were included in the quantification, they did not alter the results based on analysis of the 52 KD band alone.

Capillary rarefaction, which refers to the reduced density of capillaries, contributes to cerebral hypoperfusion, one of the prominent pathological changes in the brains of AD patients [40] and mouse models of AD [41,42]. We examined the capillary densities in our animals using Tomato-lectin staining of the brain. Compared to WT animals, the capillary area per field was significantly lower in both the hippocampus and cerebral cortex of AD rats, especially in areas near the Aβ plaques (note, Tomato-lectin partially stains Aβ plaques due to increased vascularity) (Figure 4A–C). Long-term TPPU treatment of the AD rats attenuated capillary loss in both the cerebral cortex and the CA3 region of the hippocampus. The expression of the capillary pericyte protein markers, desmin and α-smooth muscle actin (α-SMA) were also assessed to confirm this effect [43,44,45]. The expression of desmin protein was significantly reduced in the brain of AD rats, and TPPU treatment increased the expression of desmin (Figure 4D,E). The expression of α-SMA was not altered in the brains of AD rats. However, long-term TPPU treatment increased the expression of α-SMA relative in AD rats versus controls (Figure 4F,G).

### 2.5. TPPU Improves Blood–Brain Barrier (BBB) Leakage in AD Rats

**Figure 5:**
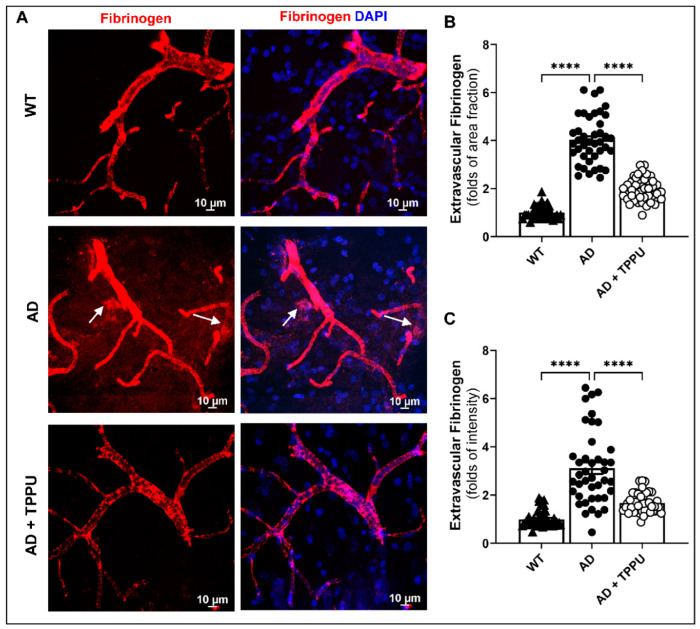
TPPU improves blood–brain barrier (BBB) leakage in AD rats. (**A**) Representative images of penetrating arterioles and capillaries co-immunostained for fibrinogen and DAPI in the cortex of 9–10-month-old Fischer 344 (WT) (n = 6), APP/PS1 TgF344-AD (AD) (n = 6), and AD treated with TPPU (AD + TPPU) (n = 6) rats, Arrows indicate areas of leakage of fibrinogen surrounding the vessels. (**B**) Comparison of the fibrinogen positive area fraction in extravascular fields. (**C**) Comparison of the fibrinogen staining intensity within and surrounding vessels and capillaries. The values in individual animals were normalized to the average value in the WT group. Data are presented as means ± SEM. **** *p* < 0.0001.

We recently reported that the myogenic response of the middle cerebral artery and PA and autoregulation of CBF were impaired in the APP/PS1 TgF344-AD rats. Long-term administration of TPPU restored the myogenic response and improved autoregulation of CBF and cognitive function in AD rats [34]. Impaired autoregulation is associated with increased pressure transmission to downstream capillaries, resulting in capillary distention and rarefaction, endothelial dysfunction, and BBB leakage that promotes cerebral inflammation and neurodegeneration [46]. The effect of TPPU on BBB integrity was explored by measuring the extravascularization of fibrinogen in the brains of AD rats (Figure 5). Areas of leakage from capillaries (white arrows) and the ratios of the intensity of fibrinogen staining within versus the area surrounding capillaries were elevated in the cortex of AD versus WT rats. The area of fibrinogen leakage and the fibrinogen staining intensity ratio within versus outside the capillaries were markedly reduced in AD rats treated with TPPU.

### 2.6. TPPU Attenuates Astrogliosis and Microgliosis in AD Rats

**Figure 6:**
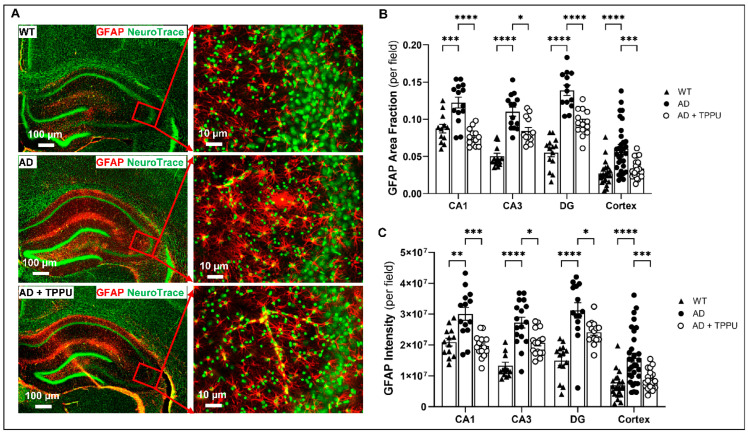

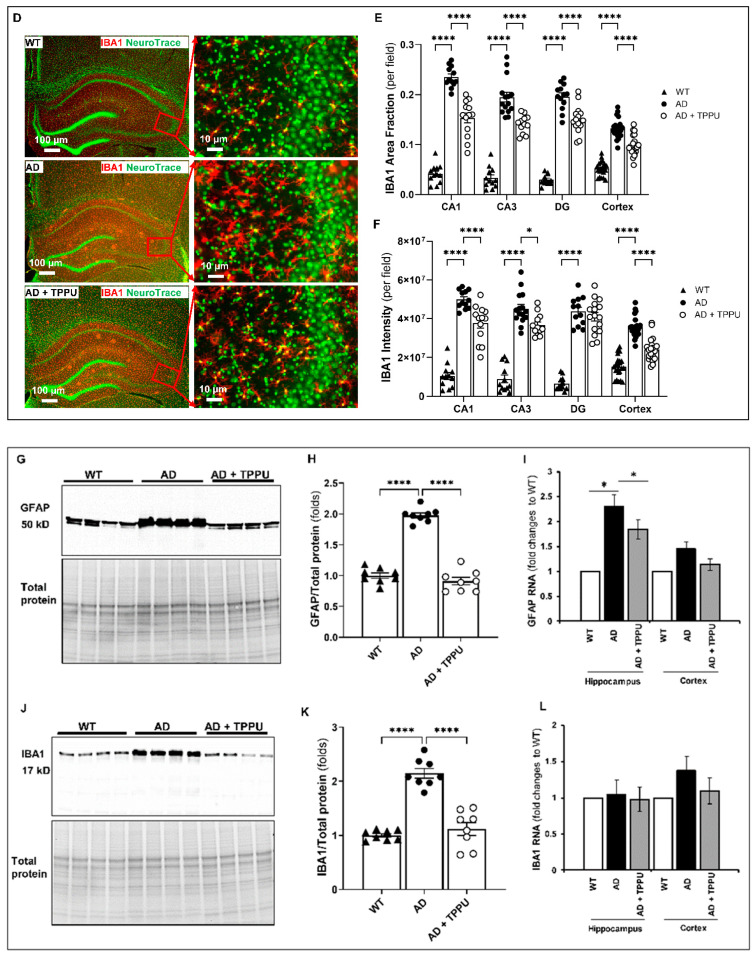
TPPU attenuates astrogliosis and microgliosis in AD rats. (**A**) Left panel: representative images of co-immunostaining for astrocytes with Glial Fibrillary Acidic Protein (GFAP) and neurons with Green Fluorescent NeuroTrace in the hippocampus and cortex of 9–10-month-old Fischer 344 (WT) (n = 5), APP/PS1 TgF344-AD (AD) (n = 6), and AD treated with TPPU for 3 months (AD + TPPU) (n = 6) rats. Right panel: High magnification images of the hippocampal CA3 region showing increased activated astrocytes in AD rats and attenuated activation in the TPPU-treated animals. (**B**,**C**) Quantification of GFAP area fraction per field and GFAP mean intensity per field in the CA1, CA3, and dentate gyrus (DG) regions of the hippocampus and cerebral cortex. (**D**) Left panel: representative images of co-immunostaining for microglia with Ionized calcium-binding adaptor molecule 1 (IBA1) and neurons with Green Fluorescent NeuroTrace in hippocampus and cortex of 9–10-month-old WT (n = 5), AD (n = 6), and AD + TPPU (n = 6) rats. The right panel presents a higher magnification of the hippocampal CA3 region, showing marked microglia activation in AD rats and attenuated activation in the TPPU-treated animals. (**E**,**F**) Quantification of the IBA1 area fraction per field and IBA1 mean intensity per field in the CA1, CA3, and DG of the hippocampus and the cerebral cortex. (**G**,**J**) Representative Western blot showing the expression GFAP and IBA1 in the brains of 9–10-month-old WT (n = 6), AD (n = 6), and AD + TPPU (n = 6) rats. (**H**,**K**) Quantification of GFAP/total protein and IBA1/total protein ratios in the Western blots. The expression of the target proteins was normalized by the sum of the total protein detected per lane on the stain-free gel. (**I**,**L**) Comparison of RNA expression of GFAP and IBA1. The values in individual animals were normalized to the average values in the WT group. Data are presented as means ± SEM. * *p* < 0.05, ** *p* < 0.01, *** *p* < 0.001, **** *p* < 0.0001.

Aβ is taken up and activates astrocytes and microglia, releasing proinflammatory mediators that reduce CBF and promote BBB leakage, inflammation, and neurodegeneration [20]. The effects of TPPU on the expression of the astrocyte marker glial fibrillary acidic protein (GFAP) and the microglia marker ionized calcium-binding adaptor molecular 1 (IBA1) were compared in AD and WT rats using RNA sequencing, Western blotting, and immunohistochemistry (IHC) (Figure 6). The area of expression of GFAP and intensity per field were significantly higher in AD brains compared to WT, indicating activation of astrocytes, characterized by the increased volume and number of processes. Long-term TPPU treatment significantly reduced the activation of astrocytes (Figure 6A–C), which was also confirmed by the Western blot analysis (Figure 6G,H). Interestingly, the expression of *GFAP* mRNA was 2.3-fold higher in the hippocampus of AD versus F344 control rats. Long-term TPPU treatment of AD rats reduced *GFAP* mRNA expression (Figure 6I). The expression of the microglial marker IBA1 was also markedly enhanced in the brains of AD rats compared to controls. Long-term TPPU treatment reduced microglial activation in the AD rats (Figure 6D–F). This was confirmed in the Western blot analysis of the expression of IBA1 protein (Figure 6J,K). However, we did not detect signicant changes in *IBA1* mRNA expression in either the cortex or hippocampus (Figure 6L). Together, these results indicate that long-term administration of TPPU reduces the activation of both astrocytes and microglia in AD rats. The effect of TPPU was greater on astrocytes than microglia, which fits with previous reports that astrocytes avidly accumulate and clear amyloid proteins.

### 2.7. TPPU Improves Synaptic Deficits in AD Rats

**Figure 7:**
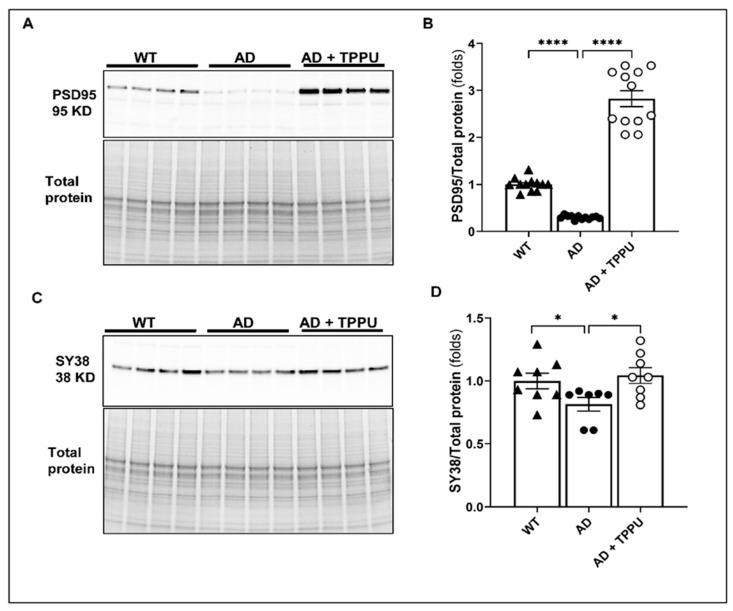
TPPU improves synaptic deficits in AD rats. (**A**,**C**) Representative Western blots showing the expression of the postsynaptic density protein 95 (PSD95) and major presynaptic synaptic vesicle p38, also known as synaptophysin 38 (SY38), in the brains of 9–10-month-old Fischer 344 (WT) (n = 6), APP/PS1 TgF344-AD (AD) (n = 6), and AD treated with TPPU for 3 months (AD + TPPU) (n = 6) rats. (**B**,**D**) Quantification of the expression of PSD95/total protein and SY38/total protein ratios. The expression of the target proteins was normalized by the sum of the total protein detected per lane on the stain-free gel. The expression levels in individual animals were normalized to the average values in the WT group. Data are presented as means ± SEM. * *p* < 0.05, **** *p* < 0.0001.

The expression of both the postsynaptic density protein 95 (PSD95) and the presynaptic vesicle synaptophysin 38 (SY38) protein was significantly reduced in the brains of AD rats versus WT rats. Long-term TPPU administration enhanced the expression of both in AD rats (Figure 7A–D). Interestingly, TPPU treatment increased PSD95 expression in the brains of AD rats to a level higher than that observed in WT rats.

### 2.8. TPPU Improves Neurodegeneration and Cognitive Impairments in AD Rats

**Figure 8:**
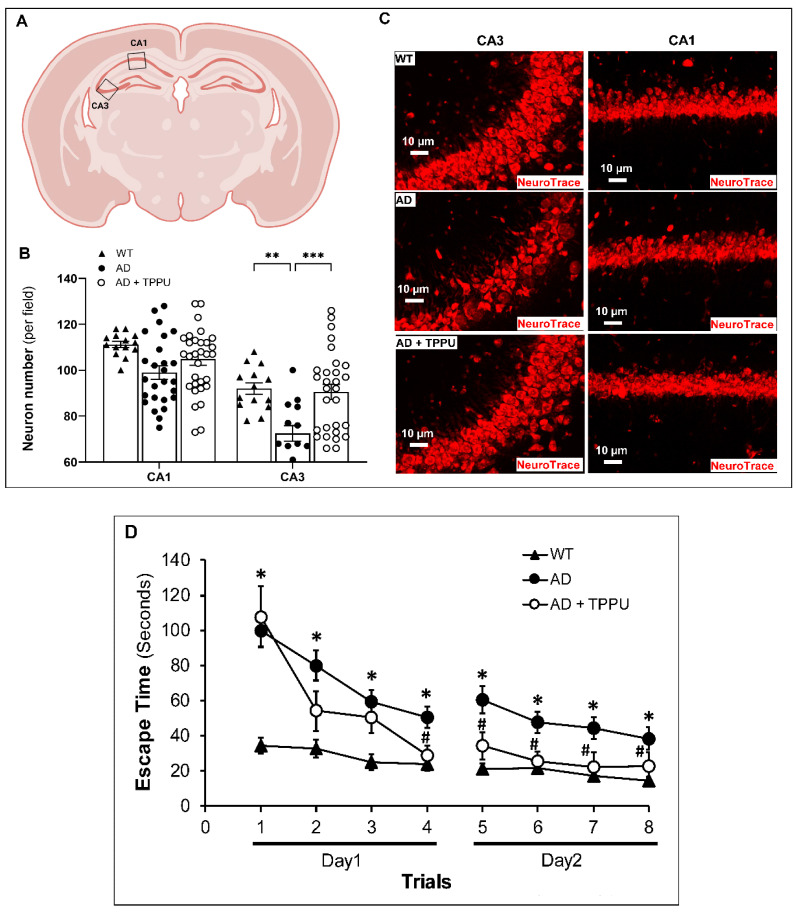
TPPU attenuates neurodegeneration and cognitive impairments in AD rats. (**A**) Schematic representation showing the regions in CA1 and CA3 of the hippocampus that were selected and studied. (**B**) Quantification of numbers of red fluorescent NeuroTrace stained neurons in the CA3 and CA1 regions of the hippocampus of 9–10-month-old Fischer 344 (WT) (n = 5), APP/PS1 TgF344-AD (AD) (n = 6), and AD treated with TPPU for 3 months (AD + TPPU) (n = 6) rats. (**C**) Representative immunostaining images for red fluorescent Nissl in the CA1 and CA3 regions of the hippocampus in the WT, AD, and AD + TPPU rats. (**D**) Results of hippocampus-dependent learning and memory test displayed as the average time (seconds) to escape in an eight-arm water maze from 9–10-month-old WT (n = 17), AD (n = 29), and AD + TPPU (n = 16) rats. Data are presented as means ± SEM. * from (**D**) indicates *p* < 0.05 from the corresponding values in AD rats compared to WT counterparts, # from (**D**) indicates *p* < 0.05 from the corresponding values in AD + TPPU rats to AD rats without treatment, ** *p* < 0.01, *** *p* < 0.001.

Neuronal numbers were markedly reduced in the CA3 region of the hippocampus in AD rats compared to WT rats (Figure 8A–C). The neurons in the CA3 region of the hippocampus of AD rats exhibited cell body shrinkage and apoptotic nuclei. TPPU attenuated neuronal loss and preserved the morphology of the neurons in this region. Neuronal number and morphology were not significantly different in the CA1 region in the brains of AD and WT rats, and TPPU had no significant effect (Figure 8A–C). We hypothesized that improved capillary density and preserved functional hyperemic responses that might have prevented transient focal cerebral ischemia and decreased activation of microglia and astrocytes and inflammation may contribute to the neuroprotective effects of TPPU in AD rats. Consistent with this hypothesis, we found that learning (day 1, escape time) and memory (day 2, escape time) were impaired in AD rats versus F344 controls in the 8-arm water maze test (Figure 8D). Three months of TPPU treatment significantly improved learning (day 1, increased slope) and memory (day 2, lower escape time) (Figure 8D).

### 2.9. Transcriptome Analysis

**Figure 9:**
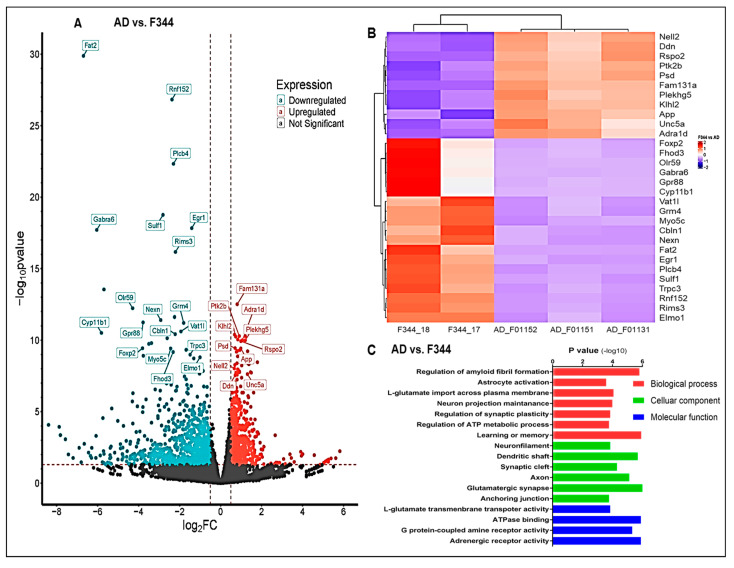

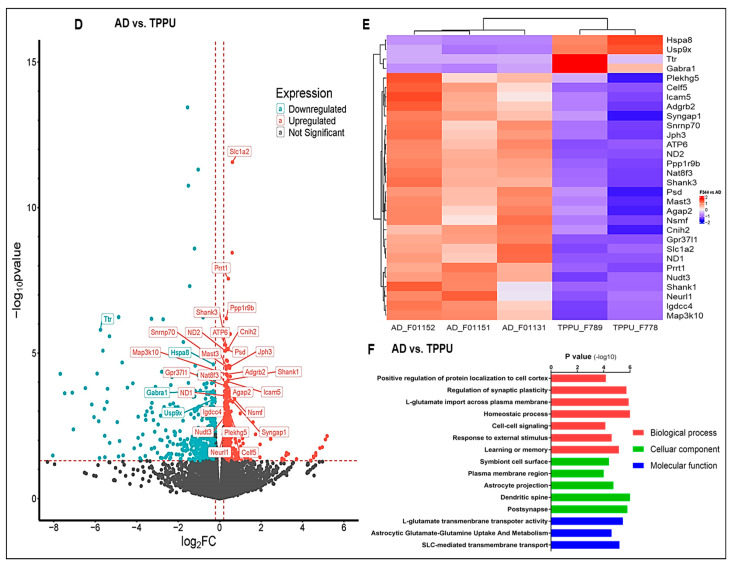
Transcriptome analysis. (**A**,**D**) Volcano plots show differentially expressed genes in the hippocampus between AD and F344 control rats (**A**) and between AD and AD rats treated with TPPU for 3 months (**D**). The x-axis presents the log2 fold change in gene expression, and the y-axis presents the −log10 *p* values. Genes with a log2 fold change> 1 and an FDR-adjusted *p*-value < 0.05 are highlighted in red (upregulated) or blue (downregulated). (**B**,**E**) Presents Heatmaps of the log2 fold changes in the top 30 differentially expressed genes in the hippocampus based on *p* values in panels (**A**,**D**). (**C**,**F**) GO analysis of the significantly altered biological processes, cellular components, and molecular function in the hippocampal region between AD and F344 control rats (**C**) and between AD and AD rats treated with TPPU (**F**).

An RNA sequencing study was performed to determine some of the mechanisms associated with the beneficial effects of TPPU in AD. A total of 1565 differentially expressed genes were detected in the hippocampus of WT versus AD rats (Figure 9A). The top 30 differentially expressed genes are presented in a heatmap (Figure 9B). Downregulation of *Rims3* (regulating synaptic membrane exocytosis 3), *Gabra6* (gamma-aminobutyric acid type A receptor subunit alpha6), *Grm4* (glutamate metabotropic receptor 4) and the upregulation of *PTK2B* (Protein Tyrosine Kinase 2 Beta), *NELL2* (Neural EGFL Like 2) in AD animals versus controls, suggest synaptic dysfunction in AD rats. The GO analysis revealed increased amyloid fibril formation, astrocyte activation, and dysregulation of glutamate reuptake pathway, synaptic plasticity, ATP production, and neurodegeneration in AD rats (Figure 9C).

In TPPU-treated AD rats, 295 genes were differently expressed in the hippocampus versus the untreated AD rats (Figure 9D). The top 30 differentially expressed genes (Figure 9E) in AD versus TPPU treated AD rats suggest an improvement in the control of the release of the excitatory neurotransmitter glutamate (higher expression of *SLCA2*, solute carrier family 1 in AD rats), a reduction in ATPase activity (reduced expression of *HSPA8*, Heat Shock Protein Family A Member 8) in TPPU treated rats, improvements in Aβ clearance, nerve regeneration pathways, and glucose homeostasis (upregulation of *TTR* (Transthyretin) and downregulation of *AGAP2* (ArfGAP With GTPase Domain, Ankyrin Repeat And PH Domain 2), reductions in the expression of inflammatory pathways (downregulation of *ICAM5*, Intercellular Adhesion Molecule 5), a decrease in mitochondrial oxidative stress (reduced *ND1 and ND2,* NADH dehydrogenase subunit 1 and 2), enhanced angiogenesis (reduced A*DGRB2*, Adhesion G Protein-Coupled Receptor B2) in the TPPU-treated than the AD rats. Additionally, the GO analysis (Figure 9F) suggested beneficial effects of TPPU on the expression of genes involved in maintaining synaptic function, improving glutamate reuptake, neural/astrocyte connectivity, cell–cell signaling, and learning or memory in AD rats.

## 3. Discussion

We previously reported that the expression of Aβ is elevated in the brain and capillary endothelial cells as early as 4 months of age in hAPP/PS1 TgF344-AD rats. This was associated with decreased expression of the Kir2.1 channel in cerebral capillaries, loss of NVC-coupled dilator response of parenchymal arterioles to potassium in vitro, an impaired myogenic response of cerebral arterioles in vitro, and autoregulation of CBF in vivo two months before the first appearance of amyloid plaques and the loss of cognitive function [37,47]. These alterations recapitulate many of the early cerebral vascular changes seen in AD patients, which are not seen in the 5x familial Alzheimer’s disease (FAD) transgenic mouse model, in which the overexpression of APP is targeted to neurons by the Thy1 promoter [12,48,49]. More recently, we found that TPPU restored the myogenic response and autoregulation of CBF and opposed the loss of cognitive function in hAPP/PS1 TgF344-AD rats [34]. However, the mechanisms linking the improvements in the myogenic response and autoregulation of CBF to the neuroprotective actions of sEH inhibition in AD remain to be determined. Thus, the present study investigated the effects of the sEH inhibitor TPPU on amyloid accumulation, NVC, BBB integrity, neuroinflammation, the loss of neurons, and cognitive function in the hAPP/PS1 TgF344-AD rats. An RNA sequence study and GO analysis were also performed to explore the potential mechanisms involved.

EETs and other epoxy-fatty acids (EpFAs) are cytochrome P450 metabolites of AA and other unsaturated fatty acids. They are potent vasodilators that resolve inflammation and oxidative stress [31,32]. Unfortunately, EETs and other EpFAs are rapidly converted by sEH to inactive, and sometimes proinflammatory diols, which limits their beneficial actions [24,25]. They are also avidly re-esterified into neutral and membrane phospholipids [50,51]. Upregulation of sEH has been reported in the brains of AD patients, 5xFAD and APP/PS1 transgenic mouse and rat models of AD [26,27,28]. In the present study, we observed a marked elevation in the expression of sEH protein in our APP/PS1 TgF344-AD rats compared to healthy age-matched F344 controls. Previous studies indicated that astrocytes are the predominant cell type expressing sEH in the brain of mouse models of AD [26,27]. Additionally, sEH is expressed in the vasculature [31,32]. Therefore, we explored the effects of TPPU on cerebrovascular function and neuroinflammation in our AD rat model. The functional hyperemic response to whisker stimulation was attenuated in AD rats, and chronic administration of TPPU normalized this response. The improvement in NVC was associated with enhanced endothelial nitric oxide synthetase expression. Compared to untreated AD rats, administration of TPPU reduced capillary rarefaction, BBB leakage, and astrocyte and microglia activation. TPPU also increased the expression of pre- and postsynaptic proteins and reduced the loss of hippocampal neurons and cognitive impairments in AD rats, which was confirmed by the transcriptome and GO analysis.

The Aβ cascade plays a critical role in AD pathologies [52]. We compared the cerebral Aβ burden between 6-month-old AD rats, 9–10-month-old AD rats, and 9–10-month-old AD rats treated with TPPU. We observed predominant Aβ accumulation in the hippocampus and cerebral cortex of 9–10-month-old AD rats and that long-term TPPU treatment reduced the size and number of amyloid plaques. TPPU did not affect the expression of *APP* or *PS1* mRNA, suggesting that it does not alter Aβ production, which is consistent with the results of a previous study [27]. LRP1, RAGE, and p-glycoprotein are the primary receptors and transporters regulating brain amyloid accumulation [36]. LRP1 and p-glycoprotein (ABCB1) proteins in cerebral endothelial cells transport Aβ from the brain to the blood, whereas RAGE binds Aβ in the brain parenchyma and promotes aggregation and deposition. The expression of LRP1 and RAGE proteins were upregulated in the brains of AD rats. The expression of *p-glycoprotein/ABCB1* mRNA was also elevated in the hippocampus of AD rats. Treatment with TPPU enhanced the expression of the *ABCB1* transporter. These results suggest that the reduction in amyloid accumulation in the brains of TPPU-treated rats was most likely due to enhanced clearance of Aβ due to reduced capillary rarefaction, endothelial damage, and increased expression of the transporters. Microglia and astrocytes also play an essential role in regulating amyloid accumulation in the brain by engulfing and degrading soluble Aβ [20]. These glial cells are less efficient in clearing amyloid [20] when activated. Thus, TPPU may also enhance the clearance of Aβ by reducing glial cell activation and inflammation.

In the present study, the functional hyperemia response to whisker stimulation was impaired in AD rats, and TPPU administration normalized this response. The functional hyperemic response is mediated by the precise coordination of NVC, which consists of neurons, astrocytes, and vasculature (endothelial cells, SMCs, and pericytes) [14]. In response to neuronal activation, neurons and adjacent astrocytes release vasoactive agents to relax upstream vessels and pericytes to increase local CBF. One important neurotransmitter is glutamate, which is released by neuronal synapses and acts on NDMA receptors in astrocytes and neurons to increase calcium influx, which triggers multiple events that increase CBF. The rise in calcium in neurons and astrocytes activates phospholipase A2 and D2 (PLA2/PLD2) to release AA and other fatty acids. AA is converted into prostaglandin E_2_ by COX1 and EETs by cytochrome P450 enzymes, respectively. Other unsaturated fatty acids are converted by cytochrome P450 enzymes to epoxy-fatty acids (EpFAs). PGE_2_ acts on receptors on vascular SMCs to promote vasodilation. EETs activate the large-conductance calcium-activated potassium (BK) channel in vascular SMCs, causing potassium efflux, cell hyperpolarization, and relaxation [14]. Some EpFAs may have similar effects. Opening of BK channels in astrocytes and activation of neurons enhance potassium efflux. Elevated extracellular K+ opens the Kir2.1 channel on vascular SMCs and endothelial cells, resulting in hyperpolarization and vasodilation [53,54,55]. ATP released by activated astrocytes also acts on purinergic (P2Y1) receptors on endothelial cells, increasing NO release that contributes to vasodilation [38]. Our RNA sequencing, IHC, and Western blot findings all support the idea that activated astrocytes play a key role in the loss of functional hyperemia, transient ischemia, cerebral inflammation, and loss of cognitive function in the APP/PS1 TgF344-AD rats. Since sEH is highly expressed in astrocytes, it is reasonable to suggest that the long-term administration of TPPU preserved astrocyte function and improved NVC and the ability to redistribute CBF to activated neurons in AD rats. Additionally, restoring normal astrocyte function prevents the accumulation of proinflammatory factors around vasculature, further facilitating an improvement of cerebral hemodynamics. Glutamate also acts on N-methyl-D-aspartate receptors (NMDARs) in neuronal synapses, increasing nNOS and PGE_2_ to facilitate vasodilation during functional hyperemia [56].

As expected, we also found a significant loss of pre- and post-synaptic proteins in the AD rats. TPPU enhanced the expression of the post-synaptic protein, which likely contributed to its neuroprotective actions in AD. The therapeutic effects of TPPU may also extend to the vasculature itself, where it appears to improve endothelial function [34].

A very recent study revealed that microglia and border-associated macrophages contribute to NVC through CD39-initiated hydrolysis of ATP, followed by CD73 or tissue-nonspecific alkaline phosphatase-mediated hydrolysis to form adenosine, leading to vasodilation [57]. We also observed a marked reduction in microgliosis in TPPU-treated AD rats, which might partially contribute to improved functional hyperemia.

We found that capillary density was reduced in the brains of AD rats, especially near amyloid plaques. TPPU treatment enhanced capillary density in the AD rats. One possible reason may be that inhibition of sEH increases levels of EETs and EpFAs, which reduces neuroinflammation and stimulates angiogenesis. Our RNA sequencing study results indicated increased expression of genes associated with angiogenesis and downregulation of genes associated with vascular inflammation in the TPPU-treated AD rats.

BBB disruption is associated with cerebral inflammation, neurodegeneration and loss of cognitive function in AD. In the present study, we observed BBB leakage of fibrinogen in the brains of AD rats, whereas no leakage was seen in the brains of the WT controls. We previously proposed that the impaired myogenic response of the cerebral microcirculation and autoregulation of CBF in AD rats increases the transmission of pressure and distends downstream capillaries, which damages the endothelium, associated astrocytic foot process and pericytes and contributes to BBB leakage in AD rats. Additionally, amyloid accumulation around penetrating arterioles increases vascular inflammation and oxidative stress and contributes to the breakdown of the BBB in AD. The reduction in BBB leakage in AD rats treated with TPPU was likely due to improved myogenic response and autoregulation of CBF, which decreased cerebral inflammation and capillary loss.

The activation of astrocytes and microglia plays a central role in AD neurodegeneration. Activated astrocytes and microglia release proinflammatory cytokines that impair vascular and synaptic function and facilitate neurodegeneration [20]. Additionally, activation of astrocytes impairs glutamate reuptake, which enhances excitotoxicity-induced neuronal damage and cognitive dysfunction. In the present study, the TPPU mitigated the activation of astrocytes and microglia, which broke the vicious cycle between amyloid accumulation, astrogliosis, microgliosis, inflammation, and neurodegeneration in AD rats. As a result, TPPU treatment protected neuronal synapses, the loss of hippocampal neurons, and preserved cognitive function in AD rats.

## 4. Materials and Methods

### 4.1. Experimental Animals and Study Design

Experiments were conducted on age-matched male hAPP/PS1 TgF344-AD rat model of AD and Fischer 344 (F344) wild-type (WT) controls. The TgF344-AD rats overexpress mutant human APP and PS1 proteins on the F344 rat genetic background and recapitulate all of the AD-related pathologies, including amyloid plaque formation, tauopathy, neuroinflammation, cerebrovascular dysfunction, neurodegeneration, and cognitive impairments [34,35,58]. We have previously reported that this AD rat model develops impaired cerebral hemodynamics at 6 months [37,47]. Therefore, we started TPPU treatment (1 mg/kg body weight/day) at the age of 6–7 months for three months. TPPU was dissolved in 1% PEG400 and then diluted to a final concentration in the drinking water [59]. Since our previous results indicated that TPPU or vehicle treatment did not affect physiological parameters or vascular function in F344 WT rats [34], we studied its effects in three experimental groups: (1) WT F344 rats (WT, no treatment), (2) TgF344-AD rats (AD, no treatment), and (3) AD rats treated with TPPU (AD + TPPU). All animals were housed on a 12 h:12 h light/dark cycle with free access to food. All protocols were approved by the Institutional Animal Care and Use Committee of the University of Mississippi Medical Center and were conducted following the National Institutes of Health Animal Care and Use guidelines.

### 4.2. Laser Speckle Imaging of Cerebral Hemodynamics In Vivo

The rats were surgically prepared with a thinned cranial window to measure CBF using a Laser Speckle Image (LSI) system (RFLSI ZW, RWD Life Science Co, Shenzhen, China). The animals were anesthetized with ketamine (30 mg/kg, im) and Inactin (50 mg/kg, ip) and placed on a thermostatic operating table to maintain body temperature. The trachea was cannulated with a PE240 tube connected to a ventilator (SAR-830, CWE Inc., Ardmore, PA, USA) and end-tidal CO_2_ analyzer (CAPSTAR-100, CWE Inc., Ardmore, PA, USA) to maintain expired PCO_2_ of 35 mmHg. A PE50 catheter was inserted into the femoral artery to monitor mean arterial pressure (MAP). The rat’s head was secured using a stereotaxic frame (Stoelting, Wood Dale, IL, USA). A midline incision was made on the top of the head. After removing skin, aponeurosis, and periosteum, the skull was carefully thinned at coordinates 2 mm posterior and 6 mm lateral to the bregma using a low-speed drill to open a translucent closed cranial window. The LSI with RFLSI Acquisition application was used to record CBF as previously described [59]. Five regions of interest (ROIs) within the whisker somatosensory area were monitored. Baseline blood flow in these ROIs was measured after a 30 min equilibration. Then, the rats’ contralateral whiskers were stimulated for 1 min at 10 Hz, and changes in CBF in the somatosensory cortex were recorded. Following the stimulation, blood flow was recorded for 30 s as CBF returned to baseline. Three trials at 5 min intervals were recorded from each animal. The changes in CBF were expressed as the percentage of the average baseline blood flow before stimulation. The mean values ± standard error of the mean (SEM) from 5 ROIs during three trials are presented.

### 4.3. Vascular Reactivity Ex Vivo

The vascular reactivity of PAs isolated from WT and AD rats to short-term TPPU administration was assessed using our published protocol [34]. Briefly, animals were euthanized with 4% isoflurane, and the brains were collected. Intact PAs were microdissected and mounted onto glass pipettes in a myograph (Living System Instrumentation, Burlington, VT, USA). The vessels were stabilized at an intraluminal pressure of 30 mmHg for 30 min. Vehicle or TPPU (10 µM) was added to the bath, and changes in the vessel’s inner diameter were recorded over 20 min.

### 4.4. Western Blot

Rats were sacrificed with isoflurane and the brains were collected and homogenized in ice-cold RIPA buffer containing 1% protease and phosphatase inhibitors (PPI; #A32963, ThermoFisher Scientific, Chicago, IL, USA) using a ground glass homogenizer followed by homogenization using a FastPrep-24 bead-beating homogenizer (#15070599, MP Biomedicals, Santa Ana, CA, USA). The homogenate was centrifuged at 3000× *g* for 5 min followed by 110,000× *g* for 15 min at 4 °C [60]. The supernatants were collected, and protein concentrations were measured using Bio-Rad Bradford assay (#5000006, Bio-Rad, Hercules, CA, USA) with BSA as the standard. Aliquots of protein (20 µg) were separated on a 4–20% Criterion™ TGX Stain-Free™ Protein gel (#678093, Bio-Rad, Hercules, CA, USA). The total protein in each lane was determined by UV imaging of the gel with a ChemiDoc XRS+ Imager System (#1708265, Bio-Rad, Hercules, CA, USA) for normalization. Proteins in the gel were transferred to 0.2-µm nitrocellulose membranes (#1704159, Bio-Rad, Hercules, CA, USA) using a Trans-Blot Turbo Transfer System (#690BR008061, Bio-Rad, Singapore). The membranes were blocked with Odyssey Blocking Buffer (#927-40000, LI-COR, Lincoln, NE, USA) for 2 h at room temperature. After briefly rinsing with Tris-buffered saline-0.1% Tween20 (TBST), the membranes were incubated with primary antibodies overnight at 4 °C. After four washes with TBST, the membranes were incubated with horse radish peroxidase (HRP) coupled secondary antibodies for 2 h at room temperature. After another four TBST washes, the membranes were incubated with SuperSignal™ West Dura Extended Duration Substrate (#34075, ThermoFisher Scientific, Chicago, IL, USA) and imaged using the ChemiDoc XRS+ Imager System. The intensity of the target protein bands relative to the sample’s total protein signal was quantified. The primary and secondary antibodies used in this study were: anti-sEH (A-5) (sc-166961, 1:1000, Santa Cruz Biotechnology, Dallas, TX, USA), anti-LRP1 (ab92544, 1:1000, Abcam, Boston, MA, USA), anti-RAGE (ab3611, 1:1000, Abcam, Boston, MA, USA), anti-phospho-eNOS (Ser1177) (#9571, 1:1000, Cell Signaling, Danvers, MA, USA), anti-Desmin (ab15200, 1:1000, Abcam, Boston, MA, USA), anti-α-SMA (A2547, 1:1000, Millipore Sigma, Burlington, MA, USA), anti-GFAP (AB5804, 1:1000, Millipore Sigma, Burlington, MA, USA), anti-IBA1 (016-20001, 1:1000, Fujifilm, Tokyo, Japan), anti-synaptophysin (SY38) (ab8049, 1:1000, Abcam, MA), anti-post density protein 95 (PSD95) (MAB1598, 1:1000, Millipore Sigma, Burlington, MA, USA), goat anti-rabbit IgG H&L (HRP) (ab6721, 1:5000, Abcam, Boston, MA, USA) and rabbit anti-mouse IgG H&L (HRP) (ab97046, 1:5000, Abcam, Boston, MA, USA).

### 4.5. Immunohistochemistry

Freshly collected brains were fixed with 10% zinc-formalin solution for 48 h. Coronal sections (20 or 50 µm) containing the hippocampus were prepared using a Compresstone vibratome (VF-310-0Z, Precisionary Instruments, Natick, MA, USA) [37]. Free-floating sections were incubated with primary antibodies in a blocking solution containing 5% BSA and 2% Triton x-100 in phosphate-buffered saline (PBS) overnight at 4 °C. After four washes in PBS-0.1% Triton x-100 (PBST), the sections were incubated with secondary antibodies in the same blocking solution for 2 h at room temperature. The sections were immunostained with Green Fluorescent Nissl stain (NeuroTrace 500/525, N21480, 1:200, ThermoFisher Scientific, Waltham, MA, USA) to visualize neurons, anti-Glial Fibrillary Acidic Protein (GFAP) antibody (AB5804, 1:500, Sigma, Burlington, MA, USA) or anti-ionized calcium-binding adaptor molecule 1 (IBA1) antibody (019-19741, 1:500, Wako, Tokyo, Japan) and a Alexa Fluor 555 Goat Anti-Rabbit secondary antibody (A21428, 1:1000, Invitrogen, Waltham, MA, USA) to label astrocytes and microglia, Tomato Lectin DyLight 488 (L32470, 1:200, ThermoFisher Scientific, Waltham, MA, USA) for vessels, anti-MOAB antibody (M1586-100, 1:500, Biosensis, Thebarton, Australia) and an Alexa Fluor 555 secondary antibody Goat Anti-Mouse secondary antibody (A21424, 1:1000, Invitrogen, Waltham, MA, USA) to detect Aβ40 and Aβ42 as we previously published [37], and anti-fibrinogen beta chain antibody (ab1489490, 1:500, Abcam, Boston, MA, USA) to detect BBB leakage. After washing with PBST, sections were mounted on glass slides with ClearVue mounting media (4212, Epredia, Breda, The Netherlands) and coverslipped. Sections were imaged by investigators blinded to experimental treatment using a Nikon Eclipse 55i fluorescence microscope connected with a DS-FiL1 color camera (Nikon, Melville, NY, USA) or a Nikon C2 laser scanning confocal system (Nikon, Melville, NY, USA). MOAB positive area fraction and MOAB mean intensity per field in the cortex and hippocampus were quantified. The GFAP/IBA1 positive area fractions and mean intensities per field were quantified in the CA1, CA3, and DG regions of the hippocampus, as well as zones 3–5 of the cortex. The lectin positive area fraction per field was quantified to assess the capillary density. The fibrinogen area and intensity within and surrounding microvessels were quantified. Neuron numbers per field in several areas of CA1 and CA3 regions of the hippocampus were manually counted.

### 4.6. Eight-Arm Water Maze

Animals were subjected to an eight-arm water maze to test spatial learning and short-term and long-term memory, as we previously described [47]. The rats were trained to identify a marked escape platform in one of the eight arms of the water maze. At the end of training, rats were gently dried with a towel and were placed back in their home cages. During the testing phase, rats were randomly placed in one of the arms of the water maze and were allowed to explore to find the platform for 3 min. Four trials with 20 min recovery intervals were performed at 2 and 24 h post-training, and the escape times were recorded.

### 4.7. RNA Sequencing and Bioinformatic Analysis

RNA was extracted using the Pure Link RNA Mini Kit (Invitrogen) from frozen samples and assessed for quality control parameters of minimum concentration and fidelity (i.e., 18S and 28S bands, RIS > 8) through the UMMC Molecular and Genomics Core Facility (MGCF). Libraries were prepared for RNA sequencing using TruSeq mRNA Stranded Library Prep Kit (Set-A-indexes), quantified with the Qubit Fluorometer (Invitrogen), and assessed for quality and size Qiagen QIAxcel Advanced System or Agilent TapeStation. The library was sequenced using P3 (200 cycles, paired-end 100 bp) on the Illumina NextSeq 2000 platform. The run generated ~0.8 billion reads (%QC30 = 92) and was assessed for quality using FastQC [61]. The raw reads were aligned using the STAR aligner (STAR-2.7.10a_alpha_220818) [62,63] to the rat reference genome (mRatBN7) with corresponding annotation GTF files. Alignment was performed with default parameters, and uniquely mapped reads were retained to generate count data using FeatureCounts (2.0.2) [64]. Initial processing (retained genes with counts >10) and data normalization were performed using the built-in normalization methods that account for differences in library sizes and biological variability in the package DESeq2 in R 4.4.1 [64,65]. Genes with an adjusted P-value (FDR corrected) at the specified threshold (Padj < 0.05) were considered statistically significant. Volcano plots, MA-plots, heatmaps, and principal components analysis plots based on ‘regularized log’ transformation were used to visualize the results of the differential expression analysis and generated with R packages ggplot2 (v3.5.1, access date: 12 November 2024), EnhancedVolcano (v1.24.0. access date: 24 January 2025), and Heatmap (v2.22.0, access date: 24 January 2025) [66,67,68]. The R/Bioconductor packages clusterProfiler 4.12.6 [69], and gprofiler2 0.2.3 [70] were used for enrichment analysis using the list of the significant genes generated as input.

### 4.8. Statistics

Data are presented as mean values ± SEM. The significance of differences in mean values between groups in the corresponding trials in the eight-arm water maze and functional hyperemic responses were compared with a one-way analysis of variance (ANOVA) for repeated measures followed by a Holm–Sidak for preplanned comparisons. The significance of differences in other factors between the three groups was determined by one-way ANOVA followed by Holm–Sidak post hoc tests using GraphPad Prism 10 (GraphPad Software, Inc., La Jolla, CA, USA). A value of *p* < 0.05 was considered to be significant.

### 4.9. Summary and Conclusions

The present study reveals that administering an sEH inhibitor to AD rats exerts vascular protective, anti-inflammatory, and anti-amyloid effects by targeting astrocytes and the cerebral vasculature, where sEH is highly expressed. This intervention led to improved cerebral hemodynamics, reduced astrogliosis and microgliosis, enhanced synaptic integrity, improved survival of hippocampal neurons, and opposed the decline in cognitive function. The primary limitation of this study is that we did not measure the effects of TPPU on EpFAs in the brain to establish that inhibition of sEH and elevated EpFAs mediated the anti-inflammatory and neuroprotective effects of TPPU. However, previous studies have reported that the elevated expression of sEH in the 5× FAD mouse model was associated with a modest (20%) reduction in free EpFAs in the brain. TPPU restored the levels of EpFAs, suggesting that they may contribute to the anti-inflammatory and neuroprotective effects of this sEH inhibitor [27,29]. However, measuring increases in free EpFA levels in the brain may not be sufficient to reflect the effects of TPPU on sEH activity since 90% of oxylipins are esterified in neutral and membrane phospholipids and are highly susceptible to postmortem ischemia and dissection artifacts [50,51]. Additional studies are needed to show that TPPU and other sEH inhibitors increase levels of esterified EpFAs in the brain. In addition, further studies showing that inhibitors of the production and actions of EpFAs attenuate the therapeutic effects of sEH inhibitors in AD would help to establish that elevated levels of EpFAs mediate these effects and to determine the mechanisms involved.

In conclusion, the present study reveals that long-term administration of the sEH inhibitor, TPPU, reduced cerebral vascular dysfunction, BBB leakage, cerebral inflammation, accumulation of beta-amyloid and the loss of cognitive function in a human APP/PS1 transgenic rat model of Alzheimer’s disease. These results have important implications since an analog of TPPU is currently being evaluated in phase 1 and 2 clinical trials for treating neuropathic pain and Parkinson’s disease and may soon be available for testing for the treatment of Alzheimer’s disease.

## Data Availability

The analyzed data and original contributions in this study are included in the article. Further inquiries can be directed to the corresponding author. Additional raw data and images supporting the conclusions of this article will be made available by the authors upon request.
